# Case report: 10-year survival of a patient with a primary hepatic gastrointestinal stromal tumor

**DOI:** 10.3389/fonc.2022.1035824

**Published:** 2022-12-01

**Authors:** Jie Lian, Meiyan Feng, Shumei Zhang, Haibo Lu

**Affiliations:** ^1^ Department of Outpatient Chemotherapy, Harbin Medical University Cancer Hospital, Harbin, Heilongjiang, China; ^2^ Department of Tumor Pathology, Harbin Medical University Cancer Hospital, Harbin, Heilongjiang, China; ^3^ College of Information and Computer Engineering, Northeast Forestry University, Harbin, China

**Keywords:** long-term survival, primary hepatic GIST, extra-gastrointestinal stromal tumors, imatinib, sunitinib, molecular-targeted therapeutic strategies

## Abstract

**Background:**

Gastrointestinal stromal tumors (GISTs) are mesenchymal tumors of the gastrointestinal tract. Extra-gastrointestinal stromal tumors (EGISTs) predominantly arise outside the gastrointestinal tract, although primary hepatic GISTs are extremely rare. GISTs are highly aggressive; they often grow to a large size. Here, we report the 10-year survival of a patient with a primary hepatic GIST following sequential response therapy.

**Case presentation:**

A 50-year-old Chinese man complained of fatigue and slight abdominal pain, and presented with a large lump in the liver, which was detected by computed tomography (CT). He was subsequently diagnosed with a primary hepatic GIST, based on CT-guided fine needle aspiration cytology and immunohistochemistry analyses. The presence of GIST or EGIST metastases was excluded using CT, esophagogastroduodenoscopy, colonoscopy, and ultrasound. Cytological examination showed that the tumor was composed of epithelioid and spindle cells. Immunohistochemistry analysis revealed positive staining for CD117 (KIT) and DOG1, and negative staining for CD34, S-100, and α-smooth muscle actin (SMA). Following tumor ablation with argon-helium cryosurgery, the patient received imatinib mesylate for 61 months. However, this treatment was discontinued because of disease progression, at which point interventional therapy was administered once. One month later, sunitinib malate was administered for 71 months. The patient achieved long-term survival for 135 months.

**Conclusions:**

EGISTs can be easily misdiagnosed as other types of tumors because they have no specific characteristics to distinguish them during imaging examinations. However, our case study demonstrates that the long-term survival of patients with EGISTs can be achieved with molecular targeted therapy.

## Introduction

Gastrointestinal stromal tumors (GISTs) are abdominal tumors of mesenchymal origin, which are located in the gastrointestinal tract. GISTs frequently contain mutations targeting genes encoding KIT (also known as CD117) or the platelet-derived growth factor receptor alpha (PDGFRA) (1, 2). KIT is a member of the type III receptor tyrosine kinase family, which also comprises PDGFRA and platelet-derived growth factor receptor beta (PDGFRB). Binding of KIT to its ligand, the stem cell factor (SCF), results in receptor homodimerization and kinase activation, which eventually leads to cell proliferation. The current diagnostic criteria for patients with GISTs are based on cytological, histological, and immunohistochemical findings, of which the most important is the expression of CD117 (3). GISTs typically occur in the gastrointestinal tract, including the stomach, small intestine, colorectum, and esophagus (4, 5). A minor subset of GISTs is found in other areas and are called extra-gastrointestinal stromal tumors (EGISTs). There are few cases of primary hepatic EGISTs reported in the literature, and only their complete surgical resection has been shown to achieve long-term survival (6, 7). Here, we report a case of a patient with a hepatic EGIST, diagnosed by computed tomography (CT)-guided fine needle aspiration cytology (FNAC), who achieved long-term survival through sequential response therapy without surgery.

## Case description

**Figure 1:**
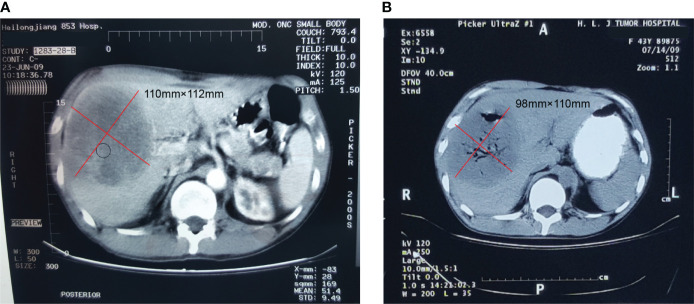
Contrast-enhanced computed tomography of the hepatic extra-gastrointestinal stromal tumor. **(A)** A 110 × 112 mm mass with solid and cystic components was observed in the right hepatic lobe, with an uneven enhancement in the arterial phase scan. **(B)** The hepatic mass was treated by argon-helium cryosurgery in July 2009, at which point the tumor was cystic and measured 98 × 110 mm.

**Figure 2:**
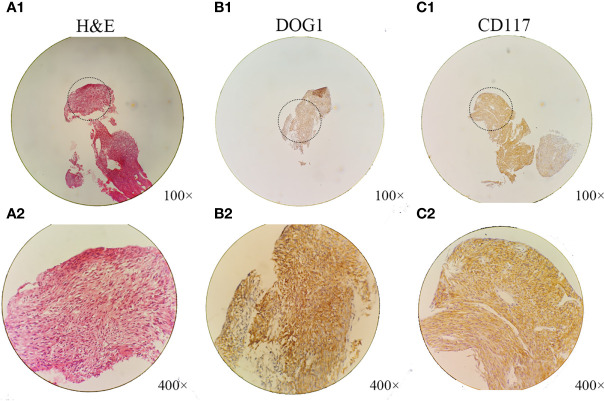
Histological and immunohistochemical analyses of the hepatic extra-gastrointestinal stromal tumor. **(A1, A2)** Microscopically, the tumor consisted of spindle cells with pleomorphic nuclei arranged into fascicles (hematoxylin–eosin stain; A1, ×100 magnification; A2, ×400 magnification). The patient’s hepatic extra-gastrointestinal stromal tumor had the following immunohistochemical staining profile: **(B1, B2)** DOG1, **(C1, C2)** CD117^+^ (**B1**, **C1**, ×100 magnification; **B2**, **C2**, ×400 magnification).

A 50-year-old Chinese man complaining of fatigue and slight abdominal pain for 1 month was admitted to Harbin Medical University Cancer Hospital (Harbin, China) on 23 June 2009. He had a history of bronchiectasis, which had not required treatment, and no family history of cancer. His physical examination was unremarkable. His levels of tumor markers, such as carbohydrate antigen (CA) 199, CA125, carcinoembryonic antigen, and α-fetoprotein, were all normal. The patient’s liver function was also normal. Dual-phase enhanced abdominal CT showed a 110 × 112 mm solid cystic lump in the right liver lobe, but no other abdominal mass (**Figure 1A**). The results of the patient’s esophagogastroduodenoscopy (EGD) and colonoscopy were unremarkable. CT-FNAC analysis revealed that the tumor was composed of spindle and epithelioid cells with high mitotic activity. Immunohistochemical staining was positive for CD117 and DOG1(**Figure 2**), but negative for CD34, S-100, and α-smooth muscle actin (SMA). GIST risk stratification is high according to the mass size, although there is no sufficient tumor area for accurate determination of mitotic rate since this is a small biopsy. The diagnosis was primary hepatic GIST.

**Figure 3:**
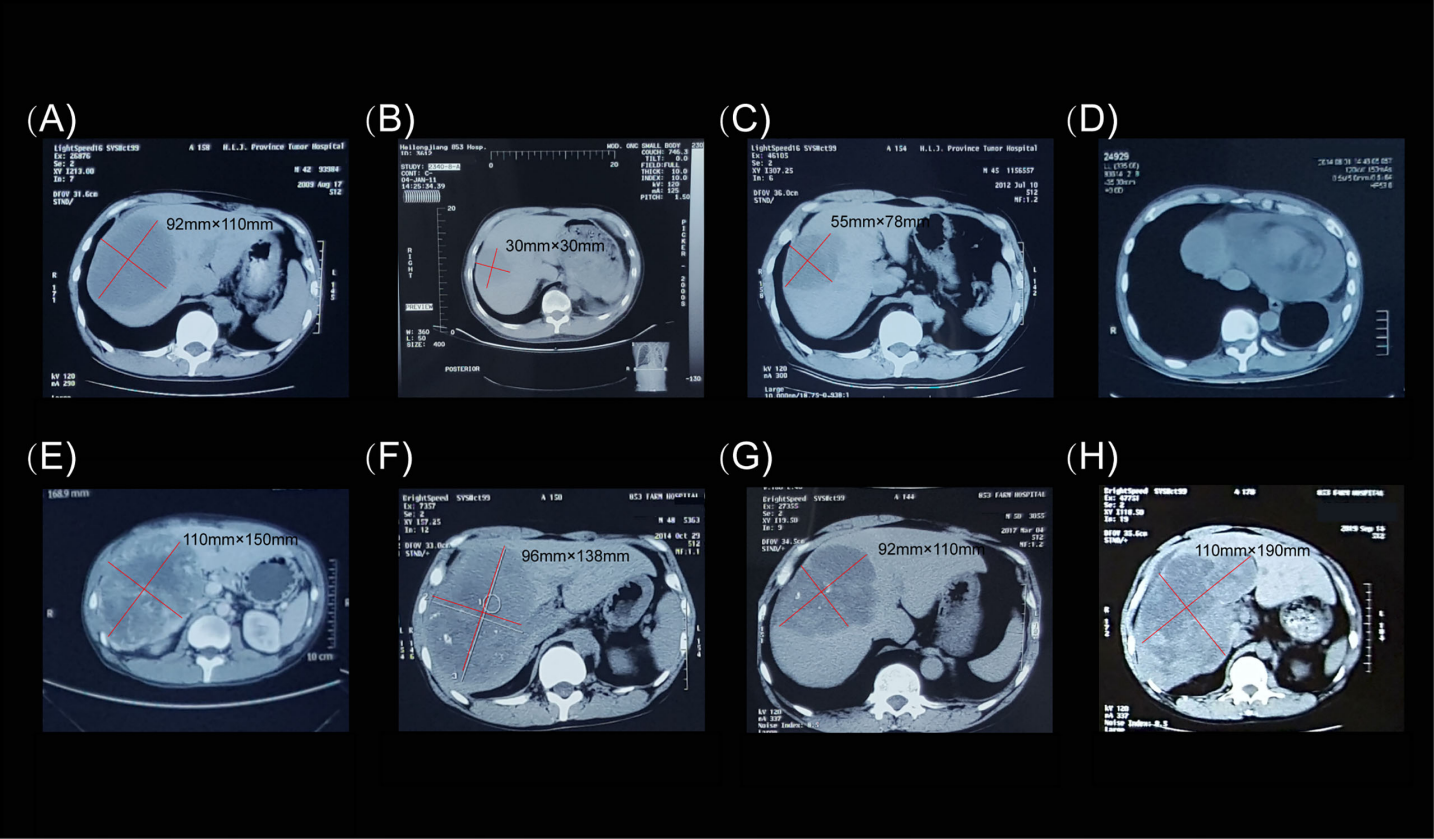
Computed tomography images of the hepatic extra-gastrointestinal stromal tumor. **(A)** In August 2009, prior to imatinib mesylate administration (400 mg once daily), the tumor mass measured 92 × 110 mm. **(B)** In January 2011, routine computed tomography examination showed that the lesion in the right hepatic lobe had shrunk to 30 × 30 mm. **(C)** In July 2012, the lesion grew to 55 × 78 mm. **(D)** Pericardial effusion was observed in August 2014. **(E)** In September 2014, the lesion measured 110 × 150 mm. **(F)** Before the patient was switched to sunitinib malate therapy (50 mg once daily on a 4/2 schedule), the tumor mass measured 96 × 138 mm. **(G)** In March 2017, the tumor was 92 × 110 mm in size. **(H)** The final computed tomography examination in October 2019 showed that the tumor mass measured 110 × 190 mm.

The hepatic mass was treated by argon-helium cryosurgery in July 2009 (**Figure 1B**). One month later, treatment with imatinib mesylate (400 mg once daily) was initiated, and the patient tolerated its adverse effects (**Figure 3A**). By January 2011, the tumor mass in the right lobe of the liver had shrunk to 30 × 30 mm, as demonstrated in a routine CT examination (**Figure 3B**). However, by July 2012, the mass had increased to 55 × 78 mm (**Figure 3C**), reaching a size of 110 × 150 mm by September 2014 (**Figure 3E**), when pericardial effusion also became visible (**Figure 3D**). After 61 months of imatinib mesylate therapy, the patient was switched to an interventional therapy consisting of transcatheter embolization with iodized oil. After a month, the patient’s tumor lesion shrank to 96 × 138 mm (**Figure 3F**). His treatment regimen was then changed to a daily dose of 50 mg of sunitinib malate; 4 weeks on, followed by 2 weeks off (a 4/2 schedule). The administration of sunitinib caused hypertension, and the patient received Plendil to lower his blood pressure.

**Figure 4:**
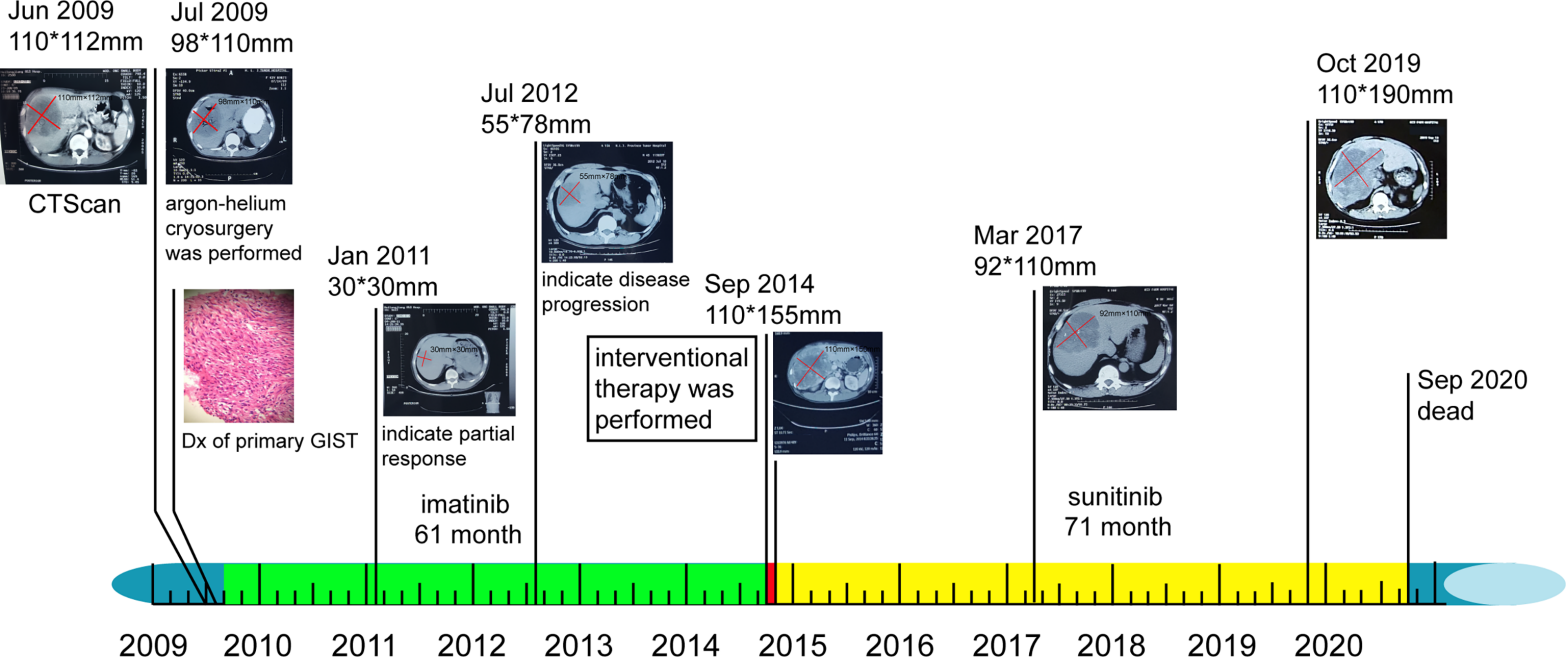
Timeline of patient’s diagnosis, treatment, and response. Colored rectangles near the timeline represent individuals’ sequential therapy durations: green, imatinib therapy; red, interventional therapy; and yellow, sunitinib therapy. Original magnification, ×400. Dx, diagnosis.

Following treatment, the lesion became stable and only cystic changes were observed during the 30-month follow-up period (**Figure 3G**). No other lesions occurred before, during, or after treatment. The patient continued to receive sunitinib. However, by October 2019, the mass had again increased to 110 × 190 mm (**Figure 3H**). We conducted telephone follow-up appointments with the patient every 3 months, but learned that he had died on 20 September 2020. The final diagnosis was primary hepatic GIST with a total survival time of 135 months. Written informed consent was obtained from the patient for the publication of this study. The authors had access to information that could identify individual participants during or after data collection. The timeline of the patient’s diagnosis, treatment, and response is shown in **Figure 4**.

## Discussion

Preoperative diagnosis is challenging in most patients with EGISTs, especially in patients with primary hepatic GISTs. The GISTs often develop into large tumors. Thus, these patients are easily misdiagnosed as having other types of cancer, such as lymphoma, malignant fibrous histiocytoma, or neurogenic tumors (6). Patients with EGISTs often have a worse prognosis than those with GISTs (8). Previously, only the complete surgical resection of primary hepatic EGISTs was able to achieve long-term patient survival (9). The treatment method for EGISTs is the same as that for stromal tumors. However, EGISTs have a higher degree of malignancy and are associated with a poor prognosis. The long-term survival of patients with EGISTs is rare.

In our case study, the patient had a large, solid, cystic mass in the right lobe of the liver. However, it was difficult to determine the nature of the tumor. Since the patient had a history of bronchiectasis and could not undergo hepatectomy, pathological diagnosis was obtained by CT-FNAC of the liver mass (10). Microscopic analysis revealed that the tumor was composed of spindle cells, some of which assumed a fence-like arrangement. In addition, there were signs of tumor hyperplasia. Biopsy specimens were small, and there was no evidence of nuclear fission across the whole field of vision. CD117 (KIT) is the most important immunohistochemical marker for the diagnosis of GIST; 94%–98% of GISTs stain positive for CD117, which is rarely expressed by other tumors. Additionally, 60%–80% of all GISTs stain positive for CD34 (11). To exclude neural tumors and smooth muscle tumors, S-100 and desmin staining, respectively, are usually recommended (3). Both the cell membrane and cytoplasm of our patient’s tumor samples were highly CD117-positive. The final primary hepatic EGIST diagnosis was based on the fact that (1) no abnormal mass was identified in any other organs except for the liver and (2) there was no evidence of other primary hepatic tumor or GIST metastases (12). Additionally, pre-, intra-, and post-operative assessment and imaging examinations, including EGD, colonoscopy, ultrasonography, and CT, revealed that the tumor was confined to the liver.

Complete surgical resection with a microscopic negative margin is the standard treatment for both GISTs and primary non-metastatic EGISTs. Peritoneal or hepatic metastases of GISTs can also be managed with localized methods such as radiofrequency ablation or chemoembolization (13). In the present case, the hepatic mass was treated with argon-helium cryosurgery in July 2009, because the patient had a history of bronchiectasis and could not undergo surgery.

Imatinib is a tyrosine kinase inhibitor (TKI) of KIT, prescribed for the treatment of GISTs and EGISTs. Imatinib can improve overall recurrence-free survival time, even in patients with advanced GISTs (14, 15). On diagnosis of an advanced GIST (unresectable, metastatic, or recurrent), therapy with imatinib mesylate should be immediately initiated, regardless of the patients’ symptoms (16). One month after argon-helium cryosurgery, our patient was prescribed the standard dose of imatinib (400 mg once daily) recommended for GIST treatment (17). The EGIST was sensitive to imatinib and gradually shrank over 18 months. However, imatinib is reported to have several side effects, such as pleural effusion (18); luckily, our patient tolerated these adverse effects.

Discontinuation of imatinib in patients demonstrating an initial favorable tumor response generally leads to rapid disease progression (19, 20). Therefore, continuous imatinib therapy is recommended, until the occurrence of intolerable adverse events or disease progression, or at the patient’s refusal. In such cases, many patients respond to the re-continuous use of imatinib, but tumor shrinkage may be smaller than was achieved prior to treatment interruption (21). Indeed, the benefits of continuing treatment despite progressive disease have been reported, depending on the available treatment alternatives (22). For our patient, imatinib administration was discontinued after 61 months when the lesion had increased in size and pericardial effusion was evident. Hepatic arterial embolization and chemoembolization have been shown to induce a radiologic response or disease stabilization in cases of imatinib-resistant GIST (23). Thus, after developing resistance to imatinib therapy, our patient underwent transcatheter embolization with iodized oil, which caused his tumor mass to shrink within a month of this interventional treatment.

Sunitinib malate is an oral multitargeted receptor TKI with selectivity for KIT and PDGFRA. Sunitinib treatment recommendations are outlined in the National Comprehensive Cancer Network Clinical Practice Guidelines in Oncology (NCCN Guidelines) and by the European Society of Medical Oncology (24, 25). The objective response rate for sunitinib was reported to be nearly 10% in the treatment of patients with GISTs following imatinib failure, and the clinical benefit rate was ~65% (26). Additionally, the median progression-free survival of patients with GISTs receiving sunitinib was 6.8 months, which was four times longer than that of the placebo arm (26). According to a previously reported dosing schedule (27), a 50-mg daily dose of sunitinib was prescribed to our patient (on a 4/2 schedule) on completion of the interventional therapy. Recent reports confirmed that sunitinib is associated with cardiac toxicity and hypothyroidism (28, 29). Therefore, careful monitoring of hypertension, cardiac function, and thyroid hormone levels is necessary during sunitinib treatment. Our patient did develop hypertension but recovered well following felodipine therapy. The patient continued receiving sunitinib for a total of 71 months and survived for a total of 135 months from the time of diagnosis until his death. It is possible that the cause of death was hemorrhage as a result of tumor rupture.

In conclusion, we report a case of long-term survival of a patient with a primary hepatic EGIST, diagnosed by CT-FNAC. Primary hepatic EGISTs are extremely rare and are difficult to diagnose preoperatively. Thus, clinicians should consider administering imatinib and sunitinib, which are already used for the treatment of GISTs, to achieve long-term survival for patients with primary hepatic EGISTs.

## Data availability statement

The original contributions presented in the study are included in the article/supplementary material. Further inquiries can be directed to the corresponding author.

## Ethics statement

The studies involving human participants were reviewed and approved by Harbin medical university cancer hospital (KY2017-19). The patients/participants provided their written informed consent to participate in this study. Written informed consent was obtained from the individual(s) for the publication of any potentially identifiable images or data included in this article.

## Author contributions

HL and JL treated the patient. JL provided the first draft of the manuscript. HL drafted and revised the manuscript. MF performed the pathological tests. SZ reviewed and revised the manuscript. All authors contributed to the article and approved the submitted version.

## Funding

This work was supported by the National Natural Science Foundation of China (Grant No. U20A20376 and 61972116), Beijing Award Foundation (Grant no. YXJL-2020-0818-0478), Wu Jieping Medical Foundation (Grant no.320.6750.2020-19-20). 

## Acknowledgments

We thank Anya Lissina, PhD, from Liwen Bianji (Edanz) (www.liwenbianji.cn) for editing the English text of a draft of this manuscript.

## Conflict of interest

The authors declare that the research was conducted in the absence of any commercial or financial relationships that could be construed as a potential conflict of interest.

## Publisher’s note

All claims expressed in this article are solely those of the authors and do not necessarily represent those of their affiliated organizations, or those of the publisher, the editors and the reviewers. Any product that may be evaluated in this article, or claim that may be made by its manufacturer, is not guaranteed or endorsed by the publisher.

## Abbreviations

GIST, gastrointestinal stromal tumor; EGIST, extra-gastrointestinal stromal tumor; CT, computed tomography; CT-FNAC, CT-guided fine needle aspiration cytology; PDGFRA, platelet-derived growth factor receptor alpha; PDGFRB, platelet-derived growth factor receptor beta; CA199, carbohydrate antigen 199; CA125, carbohydrate antigen 125; CEA, carcinoembryonic antigen; EGD, esophagogastroduodenoscopy.
